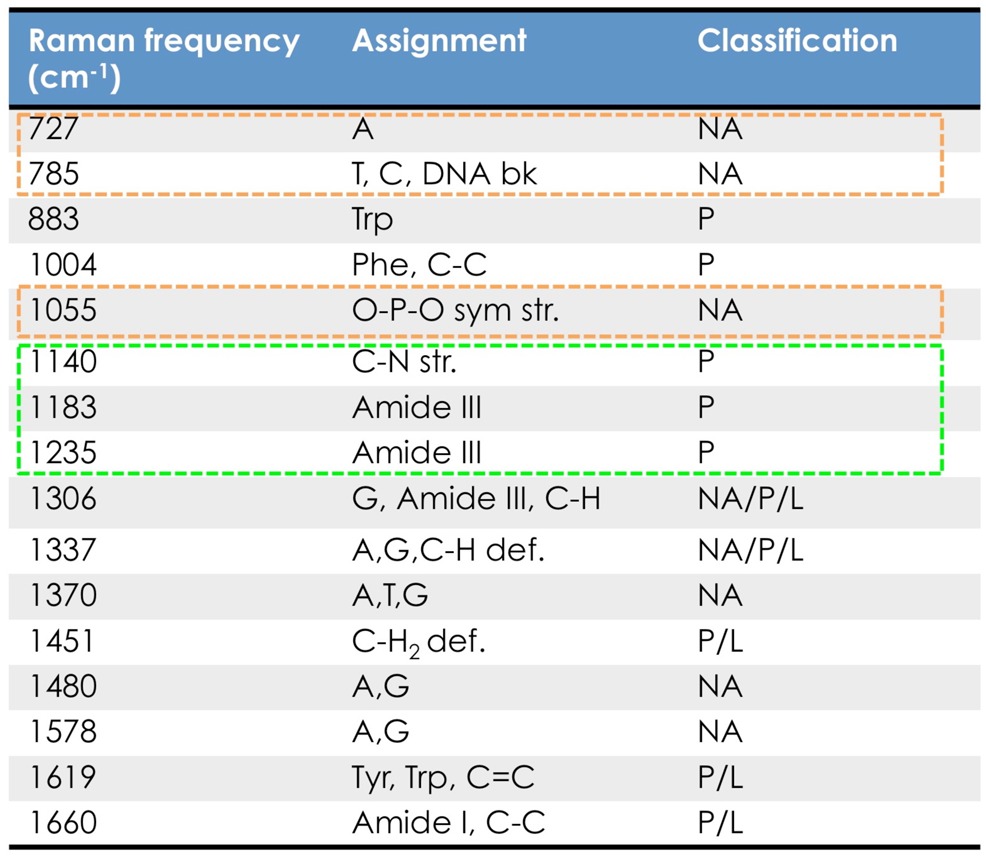# Modulated Raman Spectroscopy for Enhanced Cancer Diagnosis at the Cellular Level

**DOI:** 10.3390/s150613680

**Published:** 2015-06-11

**Authors:** Anna Chiara De Luca, Kishan Dholakia, Michael Mazilu

**Affiliations:** 1 Institute of Protein Biochemistry, National Research Council, Via P. Castellino, 111, 80131 Naples, Italy; 2 SUPA, School of Physics and Astronomy, University of St Andrews, North Haugh KY16 9SS, St Andrews, UK; E-Mails: kd1@st-andrews.ac.uk (K.D.); mm17@st-andrews.ac.uk (M.M.)

**Keywords:** Raman spectroscopy, cancer detection; cell sensor, fluorescence background

## Abstract

Raman spectroscopy is emerging as a promising and novel biophotonics tool for non-invasive, real-time diagnosis of tissue and cell abnormalities. However, the presence of a strong fluorescence background is a key issue that can detract from the use of Raman spectroscopy in routine clinical care. The review summarizes the state-of-the-art methods to remove the fluorescence background and explores recent achievements to address this issue obtained with modulated Raman spectroscopy. This innovative approach can be used to extract the Raman spectral component from the fluorescence background and improve the quality of the Raman signal. We describe the potential of modulated Raman spectroscopy as a rapid, inexpensive and accurate clinical tool to detect the presence of bladder cancer cells. Finally, in a broader context, we show how this approach can greatly enhance the sensitivity of integrated Raman spectroscopy and microfluidic systems, opening new prospects for portable higher throughput Raman cell sorting.

## 1. Introduction

An accurate diagnosis of cancer requires the identification of tumour cells and molecular precursors. This is routinely achieved by obtaining stained cell or tissue samples for subsequent analysis by a pathologist. In some clinical situations, morphological approaches are of low sensitivity or too time consuming, for instance screening urine in the case of bladder cancer [1]. Importantly, a multimodal approach is coming to the fore in a clinical setting. As an example, morphological information can be combined or supplemented with molecular information. This can enhance, or ultimately replace, standard clinical procedures, as it will deliver a higher degree of both sensitivity and specificity for disease diagnosis.

Due to its fingerprinting characteristics, Raman spectroscopy is well suited for the investigation of biological material. Raman spectroscopy is a vibrational spectroscopy technique based on the inelastic scattering of light after its interaction with a given analyte. The resultant wavelength shift can be used to detect vibrational and rotational modes of molecules present in the sample. Raman spectroscopy is a powerful tool providing details of the chemical composition and molecular structures in cells and tissues [2–7]. The onset of cancer induces chemical and structural changes in the molecular composition of the affected cell. These can include an increased nucleus-to-cytoplasm ratio, disordered chromatin, higher metabolic activity and changes in lipid and protein levels. All of these molecular changes may be recorded in the Raman spectra and can manifest themselves as the absence or presence of peaks, changes in peak intensities or peak shifts [8–11]. As these spectral changes are very specific and unique, they may determine a chemical fingerprint for the cell or tissue under investigation and indeed can be used for early diagnosis. Major advantages of Raman spectroscopy are that it is non-destructive, especially when using light in the therapeutic window (700–1100 nm). Furthermore, it does not require any labelling with extrinsic contrast-enhancing agents.

Despite the fact that Raman spectroscopy provides such important molecular information, the technique has not been incorporated into routine clinical diagnosis for several reasons. This includes the speed of acquisition, ease of potential implementation, cost and diagnostic sensitivity. A major obstacle in the development of clinical Raman applications is the strong fluorescence background, which is partially attributed to the low cross-section of Raman scattering (∼10^−30^ cm^2^). Thus, the fluorescence background partly or completely obscures the weak Raman signals, making the detection of useful spectra difficult [12]. This is especially the case for biological samples [13,14]. In turn, the presence of the fluorescence background may reduce the diagnostic accuracy and sensitivity of Raman measurements in a clinical context.

In this review, we provide a view of the state-of-the-art of the methods used for reducing/rejecting fluorescence background and enhancing Raman signals [12], such as mathematical approaches [15–26], polarization gating [27–30], time domain [31–43], frequency domain [44–47] and wavelength domain methods [48–62]. This is a contemporary and evolving field that is poised to drive Raman spectroscopy to more relevant clinical studies.

In the final part of this review, we concentrate our attention on recent results obtained by means of modulated Raman spectroscopy [63,64]. In particular, we discuss the acquisition of real-time fluorescence-free Raman spectra and show how this approach can improve the spectral quality of the Raman data by revealing very weak Raman features generally masked by the fluorescence [65,66]. To demonstrate the effectiveness of this approach, we discuss the analysis of separate spectra from normal/cancer bladder cells. We are able to identify characteristic Raman features associated with DNA, protein and lipid molecular vibrations for discriminating between the two cell types, in the absence of an interfering fluorescence background [65]. By using principal component analysis (PCA) for data classification, we demonstrate that such a modulated Raman spectroscopy approach facilitates spectral assignment and increases detection sensitivity and specificity. Further, by optimising the modulation parameters, we can achieve a complete discrimination between cancer/non-cancer bladder cells with a total acquisition time of only a few seconds [67]. Finally, we demonstrate that this modulated Raman method can robustly improve the performance, not only of bulk free-space systems, but can be applied to portable approaches (wave guide-confined Raman spectroscopy and paper microfluidics) [68,69], opening new opportunities to develop portable microfluidic devices for higher throughput Raman analysis.

## 2. Overview of Fluorescence Suppression Methods

The problem of sample fluorescence background in Raman experiments has been recognised for decades, and many different strategies have been developed to circumvent the problem.

The discrimination of out-of-focus background associated with the culture media in the case of cell or tissue analysis has been successfully realized using confocal Raman microscopy techniques [70–72] or more sophisticated phase-sensitive detection schemes [73,74]. However, it is often the sample itself that produces the fluorescence background noise, which means it cannot be spatially separated from the Raman signal.

One simple method to reduce the fluorescence is to use near-infrared (NIR) or ultraviolet (UV) excitation sources, minimizing the spectral overlap between the Raman and fluorescence spectra [75,76]. However, this option is applicable only for a relatively narrow class of molecules, as NIR excitation gives low sensitivity and UV excitation can induce sample degradation and photodamage.

Novel Raman enhancing approaches, such as SERS (surface-enhanced Raman scattering), have been used, as the fluorescence background can be quenched and the Raman signal enhanced when the analyte molecules are in the enhanced near-field of the metallic nanostructures [77]. Alternatively, some non-linear techniques, such as CARS (coherent anti-Stokes Raman scattering) [78,79] or SRS (stimulated Raman scattering) [80], generally used in imaging configurations, can enhance the Raman signal and dramatically reduce the fluorescence background.

Here, we report an overview of the methods proposed for either reducing or rejecting the fluorescence background and, thus, the direct extraction of spontaneous Raman signals. Table 1 compares the major fluorescence suppression methods.

### 2.1. Mathematical Approach

Mathematical approaches are standard techniques for fluorescence rejection. These require no setup modification and impose no limitation on sample preparation. Among these computational techniques, polynomial fitting [15–18], wavelet transformation [19,20] and derivative processing [21–23] are three major and popular background-correction algorithms in Raman spectral analysis. These approaches are applicable to the case of biomedical samples [17,20,24,25]; however, each of them is limited in certain aspects.

The polynomial approach is based on fluorescence spectrum fitting with a low-order polynomial, which is then subtracted from the Raman spectrum. This approach relies on user intervention selecting the locations of non-Raman part of the spectrum where to fit the curves. Alternatively, automated polynomial fitting methods have been introduced, but their use can be limited in high noise circumstances [15–17].

Wavelet transform methods can also be used to automate the curve fitting [19,20]. These approaches split the signals into different frequency components and then remove the low-frequency background. Wavelet background-correction algorithms suppose that the background is well separated in the transformed domain from the signal, but the real scenario does not always agree with this hypothesis. Additionally, the choice of the wavelet threshold and the proper level of baseline resolution may affect the background removal results.

First- and second-order derivative processes are based on the derivative transformation of a spectrum with subsequent rejection of the background components, which is always assumed to be lower in magnitude. This approach enhances the sharp Raman peaks [21–23]. While derivative approaches show significant computational advantages, they have the severe disadvantage in that they emphasize any high-frequency noise in the spectra. This problem can be so severe that the differentiation must be carried out as two sequential differentiation steps with smoothing after each step [22].

Hasegawa *et al.* [26] proposed the use of principal component analysis (PCA) to separate fluorescence from the Raman signal. This subtraction procedure can sometimes cause artefacts in the processed data, as it assumes that the highest signal variance is due to the fluorescence background, which may not be valid in certain applications.

Recently, an “intelligent background-correction algorithm” has been proposed by Zhang *et al.* [18] combining the wavelet method for peak detection, wavelet derivative for estimation of peak width and penalized least squares background fitting. This approach adaptively separates the spectra into peak and non-peak (background) values by setting the least squares weights to one for background and zero for peak regions. However, the application of binary-valued weights may cause some sudden changes in gradient, affecting the Raman background subtraction.

There also exist some other computational methods for fluorescence suppression, such as the asymmetric least squares method [81,82] and sensitive non-linear iterative peak (SNIP) clipping algorithms [83]. However, they are relatively less often used compared with the above major mathematical methods.

Therefore, though mathematical approaches represent the most cost-effective choice, their performance depends on the user experience and is limited, as the computational procedures can sometimes cause artefacts in the processed data, affecting the validity of the acquired biochemical information.

### 2.2. Polarization Gating

This approach, based on polarization modulation of the laser light, is another conventional method for fluorescence rejection [27–30]. The technique takes advantage of the different polarization properties of the Raman and the fluorescence signals. Indeed, Raman scattering is highly polarized, and its polarization properties depend on the excitation source while the fluorescence is totally depolarized. Therefore, by acquiring two different spectra using the orthogonal Raman excitations, it suffices to subtract the two spectra to obtain a Raman spectrum free from any fluorescence contribution. However, the method fails in several non-ideal situations, where the fluorescence may not be completely depolarized and additionally not all of the Raman features can be detected [28].

### 2.3. Time Domain Methods

**Figure 1:**
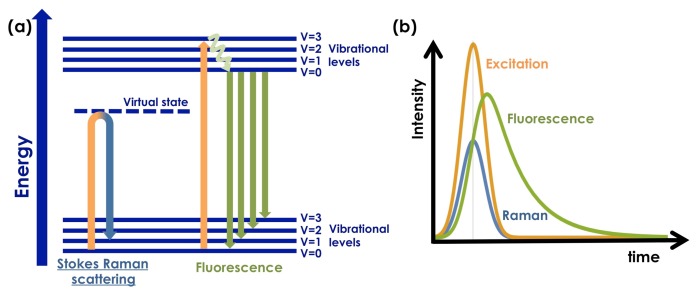
(**a**) Energy level diagram for Raman scattering and fluorescence emission; (**b**) temporal variation of excitation, Raman scattering and fluorescence emission.

Time domain methods have been employed to remove fluorescence background from Raman spectra based on the difference of lifetimes between the two processes [31–40]. The temporal profile of the Raman emission is determined by the dephasing time of the transition and is typically on the picosecond to femtosecond time scale (see Figure 1). By contrast, the intrinsic fluorescence lifetime is related to the Einstein coefficient for the emission transition. This results in lifetimes on the nanosecond to hundreds of picosecond time scale, even for very strong emitters (see Figure 1). The two orders of magnitude difference in the time scales between fluorescence emission and Raman scattering allows fluorescence to be rejected from the Raman spectrathrough time-resolved methods. Experimentally, time-resolved fluorescence rejection requires ultrashort laser pulses and fast detectors.

In 1972, Yaney [31] implemented, for the first time, a time-resolved approach using a pulsed Q-switched frequency-doubled Nd:YAG laser and a nanosecond photon-counting detection system, demonstrating an enhancement factor of 63 in the signal-to-noise ratio of the pulsed Raman signal of single-crystal strontium fluoride compared to continuous-wave Raman scattering.

Since then, a series of techniques have been used to realize short gating time in Raman measurements and to achieve a high efficiency in the fluorescence rejection [32,33]. Thus, the efficiency of the temporal fluorescence rejection is determined by the ratio between the fluorescence lifetime and the gating time of the detection, and a higher time resolution (a shorter gating time) provides higher efficiency in fluorescence rejection.

An increase in time resolution can be obtained using the optical Kerr gate approach. This method requires two crossed polarisers and a non-linear Kerr medium [34–36]. The linearly-polarized pulsed laser light interacts with the Kerr medium, inducing a polarization rotation through a third-order non-linear effect. Therefore, this rotates the polarisation of both fluorescence and Raman scattering propagating through the medium. The propagation length through the Kerr medium or the strength of anisotropy can be chosen so that the polarization of the light is transformed back to being linearly polarized, but rotated by 90° with respect to its original polarization direction. Thus, the medium acts as a transient λ/2 wave plate, and the rotated light is then transmitted through the cross-polariser. By properly synchronising the gating laser pulse, the fluorescence, which has longer lifetimes, is blocked by the polarizers. By using a Kerr gate with ∼4-ps resolution at a 1-kHz repetition rate and a CCD camera, Matousek *et al.* [35] demonstrated suppression of three orders of magnitude of the background from fluorophore with a lifetime of about 3 ns. Due to the recent advances in detector technologies, the duty cycle of the detection system could be improved by several orders of magnitude [36,41,42]. Alternatively, it is possible to use ultrafast time gated detectors, such as a fast photomultiplier [84], an intensified charge-coupled device (ICCD) [85] camera and, recently, complementary metal-oxide-semiconductor (CMOS) detectors [38], in order to reject fluorescence background [86,87]. The Kerr-gated Raman spectroscopy technique was successfully used to obtain spectra from different depths through both the prostate gland and the bladder [43]. With the help of Raman spectroscopy and Kerr gating, Prieto and co-authors demonstrated that it may be possible to pick up the spectral differences from a small focus of adenocarcinoma of the prostate gland in an otherwise benign gland and also stage the bladder cancers by assessing the base of the tumour post resection [43].

Time-resolved Raman spectroscopy is one of the most straightforward solutions to the fluorescence background problem. The main limits of this approach are the high-cost pulsed laser required for the non-linear process and the fast detector needed for gating temporal resolution.

### 2.4. Frequency Domain Methods

Rejection of fluorescence from a Raman spectrum can equivalently be realized by frequency domain methods [44]. These approaches require intensity-modulation of the excitation laser at a high frequency [46] and monitoring phases and amplitudes of the individual frequency components that comprise the time domain response [45]. Thus, frequency and time domain approaches are related to each other by the Fourier transform. Therefore, the long-lived fluorescence is demodulated, while the Raman signal can instantaneously follow the high-frequency modulated laser intensity. Two basic approaches can be used to reject fluorescence: frequency domain demodulation and phase nulling (phase quadrature). In the first case, the phase shift between the fluorescence and Raman signal is used to discriminate the two components. In phase nulling, a phase-sensitive detection based on the use of a lock-in amplifier is used to detect the modulated Raman signal. As the lock-in phase is 90° shifted compared to fluorescence phase, the fluorescence signal is completely nulled. By using a conventional photomultiplier, the enhancement achieved using the frequency domain method is higher than the one reported for a time-resolved experiment using a high-performance microchannel-plate photomultiplier [47]. Additionally, the phase nulling approach allows one to reduce the fluorescence, even in a mixture of solvents.

A disadvantage of this approach is that the experimental setup is complicated and further phase nulling requires high Raman signal levels to overcome the fluorescence noise. On the other hand, time-resolved spectroscopy has a lower fluorescence rejection capability; however, it is able to reject the fluorescence background even when the Raman signals are low comparable to the fluorescence [47].

### 2.5. Wavelength Domain Methods

By shifting the wavelength of the excitation laser, the corresponding Raman bands are also shifted while the broad fluorescence background is nearly insensitive to small (less then 1 nm) excitation wavelength changes. This property has led to various methods, such as shifted excitation Raman difference spectroscopy (SERDS) [48–51]. Shreve *et al.* [49] for the first time demonstrated that subtracting two Raman spectra, each one excited by slightly shifted laser lines, could enable the rejection of the fluorescence. This approach requires a laser source able to produce at least two slightly shifted excitation wavelengths (comparable to the full width at half-maximum of a typical Raman peak). For this aim, several laser systems have been proposed [53–59].

Mosier-Boss *et al.* [60] demonstrated a variation of this technique, where there was no shift of the laser wavelength, but a slight angle variation of the diffraction grating within the collection spectrometer to obtain two slightly spatially-shifted spectra. The main advantage of this approach is that it does not require modification of existing instrumentation. However, it has been shown that this is a not a very strong approach to remove the fluorescence [48].

Bell *et al.* [52] in 1998 proposed a similar subtracted shifted Raman spectroscopy (SSRS), where the spectra were acquired at several different closely-spaced spectrometer positions. This method is conceptually similar to SERDS, but has the distinct experimental advantage that it does not require a tunable laser source [88]. The “derivative” spectra obtained as the raw data are converted into a more recognisable and conventional form by iteratively fitting the bands to double Lorentzian functions. Osticioli *et al.* [89] proposed mathematical methods based on Fourier transform to process the SERS spectra and provide recognizable Raman signals.

SERDS technology has been successfully used in situations of biomedical interest *(in vitro* and *in vivo)* [51,61,62]. However, the use of only two or a few excitation wavelength provides a poor performance in the retrieved Raman signal. A major advance would be a simple, widely applicable method to remove the background, improving on the above-mentioned methods.

In our recent papers [63,64], we presented the theory and the implementation of a novel modulated Raman spectroscopy technique to filter out the Raman spectra from the fluorescence background through the continuous modulation of the excitation wavelength. In this way, our method allows separation of the modulated Raman peaks from the static fluorescence background with important advantages when compared to previous work using only two [49] or a few shifted excitation wavelengths [54].

In the next section, we describe this modulated Raman spectroscopy approach and the improved efficiency it may achieve for biomedical applications.

## 3. Modulated Raman Spectroscopy

### 3.1. Theoretical Background

In the wavelength-modulated Raman spectroscopy method, the excitation wavelength is changed periodically and continuously over time. The speed of this wavelength variation is slow compared to the acquisition duration, itself chosen such that the Raman peaks of interest are detectable. During the modulation of the excitation wavelength, a series of *N* spectra are acquired, denoted in the following by *S_j_*(*k*), where the subscript *j* corresponds to the *j*-th acquisition and *k* = 2π/λ to the angular wavenumber. These spectra can be seen as a superposition of two components:

(1) $$S_{j}\left(k\right)=S_{F}\left(k\right)+S_{R}\left(k+\delta k_{j}\right)$$

where *δk_j_* denotes the angular wavenumber shift of the excitation wavelength for the *j*-th acquisition. The fluorescent part is denoted by *S_F_*(*k*), while *S_R_*(*k* + *δk_j_*) spectra correspond to the Raman peaks.

To extract and distinguish between the fluorescent and Raman part of the spectra, we use PCA of the *N* spectra acquired. This approach delivers a succession of spectra, termed principal components, describing the largest variations observed between the *N* spectra. The first principal component corresponds to the largest variation. In our case, this largest variation spectra originates from the shifting of the Raman spectra *S_u_*(*k* + *δk_j_*) and delivers derivative-like Raman spectra, where the peaks are replaced by zero crossings and the fluorescence background is eliminated [64,90]. In the case of only two shifted wavenumbers (*j* = 1, 2), the first principal component is equal to the SERDS spectrum.

### 3.2. Experimental Setup

**Figure 2:**
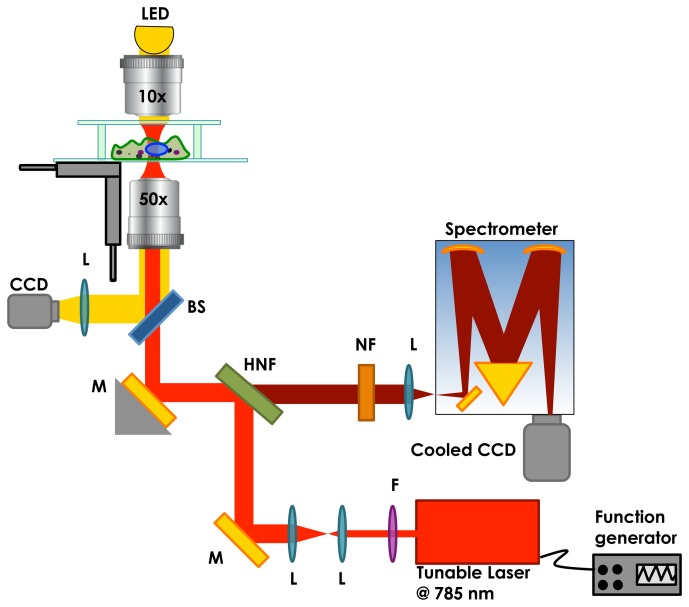
Schematic of our modulated Raman microscope system. A tunable laser at 785 nm is used to excite Raman scattering. The laser beam is introduced into an inverted microscope through a high numerical aperture objective. The scattering light from the sample is collected by the same objective, filtered by the holographic notch filter and coupled into a spectrometer equipped with a cooled CCD camera. Abbreviations: M, mirror; L, lens; F, filter; BS, beam splitter; NF, notch filter; HNF, holographic notch filter.

In Figure 2, we show a schematic of the modulated Raman microscope. Except for the laser, the setup used for the modulation technique is identical to the standard Raman microscope. The laser source was a tunable diode laser (Sacher Lasertechnik, TEC-420-0780-1000) centred at 785 nm, with a maximum power of 1 W and a total tuning range of up to 200 GHz. A waveform function generator, connected to the laser, is used to modulate the laser wavelength. The laser beam was expanded using a telescope to fill the back aperture of the objective lens. The 5-mm diameter laser beam is passed through a line filter (Semrock optic, Max line 785) and reflected from a notch filter (Semrock) into the inverted microscope. A 50 × objective lens (Olympus, oil immersion, NA = 0.95) was used to focus the laser light on the sample and to collect the backscattered photons. The scattered signal from the sample was then filtered by the same 45° notch filter, transmitting only the Raman shifted light, and imaged into the spectrometer (Shamrock SR-303i-B, Andor, Belfast, UK). A second notch filter (Semrock optics Razoredge 785) is used to suppress any remaining Rayleigh scattering. The spectrometer employed a 400-line/mm grating, blazed at 850 nm, and is equipped with a cooled CCD camera (Newton CCD, Andor, Belfast, UK) for detection of the Raman spectrum. The Raman signal was focused on the entrance slit (set at an aperture of 50 μm) of the monochromator. The entrance slit aperture together with the detector size in combination with the objective defines a cylinder of examination in the focal plane with diameter ∼0.7 μm and a depth of ∼1 μm. The spectral resolution of the system was around ∼2 cm^−1^. An LED allows white light illumination of the sample to capture transmission images on a conventional CCD camera.

### 3.3. Modulated Raman Technique and Comparison with the SERDS Method

**Figure 3:**
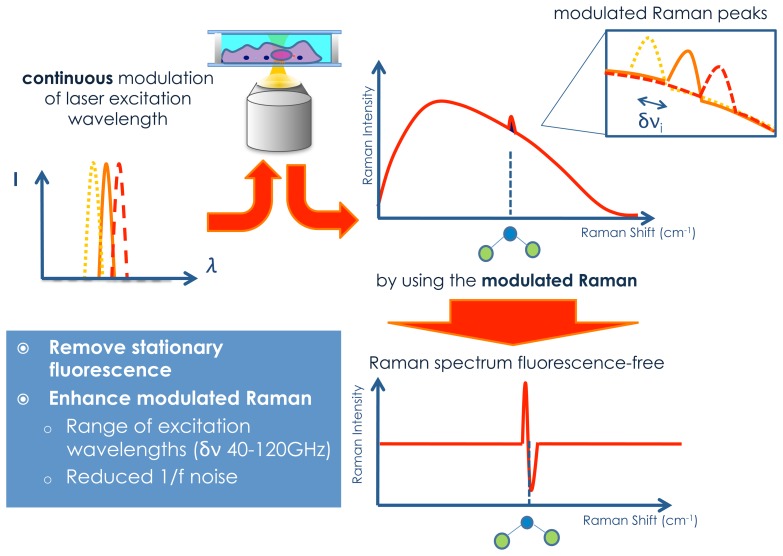
Scheme of the modulated Raman method.

Wavelength-modulated Raman spectroscopy is based on the periodic modulation of the laser excitation wavelength and the use of multi-channel lock-in detection (see Figure 3). As described in the theory section , PCA was used to obtain differential Raman spectra extracted from each set of acquired wavelength-modulated Raman signals. While this approach is conceptually similar to SERDS or single-channel wavelength modulation techniques, it offers important advantages and improvements, *i.e.*, higher signal-to-noise ratio, shorter accumulation signals and on-line access to the fluorescence-free Raman data [64].

**Figure 4:**
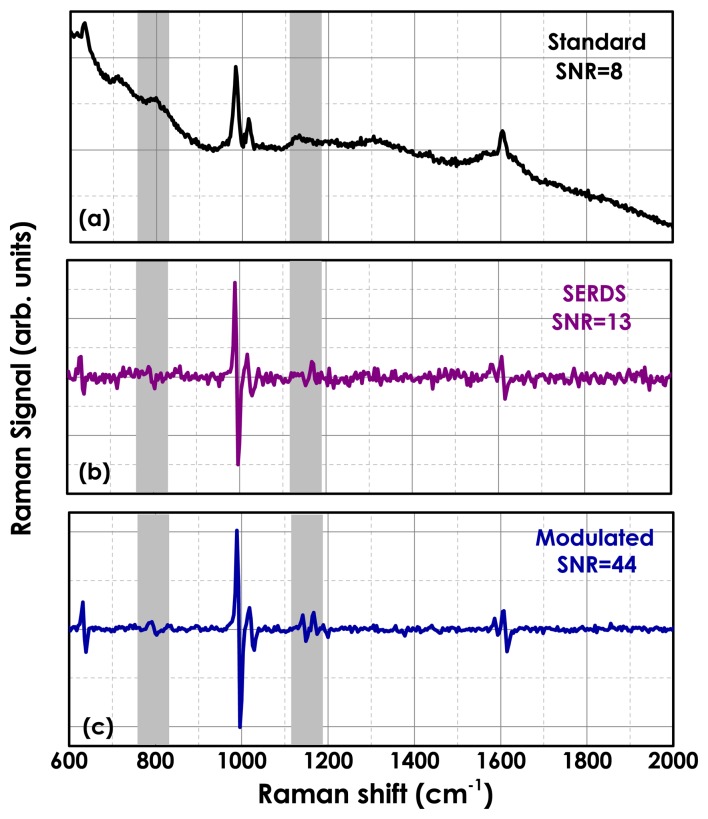
(**a**) Standard Raman spectrum of a polystyrene bead (2 μm sized) in a solution 10**^−7^** M of NIR-dye; (**b**) the shifted excitation Raman difference spectroscopy (SERDS) spectrum is obtained by acquiring only two spectra with an integration time of 5 s each at two slightly different laser wavenumbers (*δν* ∼ 40 GHz); (**c**) the modulated spectrum is acquired by modulating the Raman excitation wavelength with a frequency of *f* ∼ 1 Hz and a modulation amplitude *δν* ∼ 40 GHz. One hundred spectra are acquired with an integration time of 0.1 s each.

**Figure 5:**
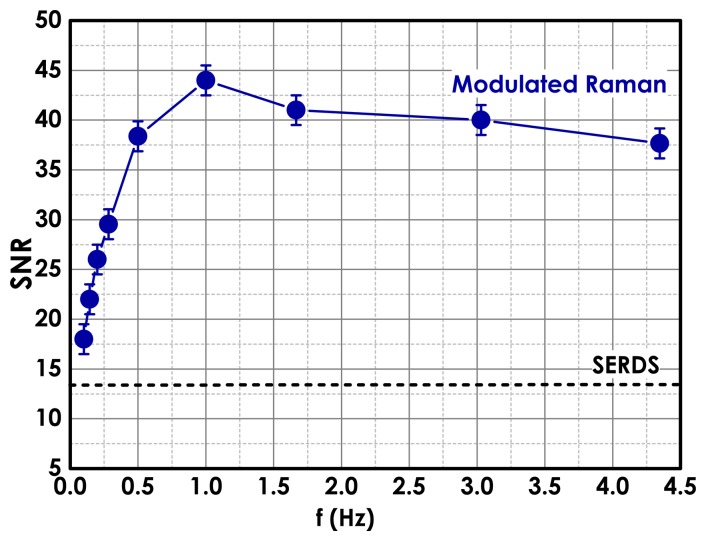
Signal-to-noise ratio (SNR) of the polystyrene Raman peak at 1001 cm^−1^ as a function of the laser wavenumber modulation rate (*f*) for modulated Raman spectra. The SNR measured by using the SERDS approach is additionally shown (dashed line).

Figure 4 shows a typical standard Raman spectrum of a polystyrene bead (2 μm sized) in a solution 10^−7^ M of NIR-dye (Fluorescent Red NIR, Sigma-Aldrich, St Louis, MO, USA). In Figure 4a, we observe the polymer Raman peaks on top of a broad fluorescence signal. The laser power on the sample was 5 mW and the integration time 10 s. In Figure 4b, we show the SERDS spectrum, obtained by acquiring only two spectra with an integration time of 5 s each at two slightly different laser wavenumbers (*δν* ∼ 40 GHz). This spectrum is free from any fluorescence contribution, but not all of the weak Raman bands are visible due to the low signal-to-noise ratio inherent in this approach. Better results are obtained by plotting the eigenvector of the PCA covariance matrix with the largest eigenvalue (first principal component, PC1), as shown in Figure 4c. The modulated spectrum is acquired by modulating the Raman excitation wavelength with a frequency of *f* ∼ 1 Hz and a wavelength oscillation amplitude equivalent to a bandwidth of *δν* ∼ 40 GHz. One hundred spectra are acquired with an integration time of 0.1 s each. In the modulated Raman spectrum, both scattering and fluorescence from the sample and spurious stray light [66] are completely suppressed thanks to the modulation of the Raman excitation wavelength and the multi-channel lock-in detection. Additionally, the use of a continuously-modulated wavelength in our method as opposed to the use of only two (or few) excitation wavelengths in SERDS helps further in the reconstruction of the Raman signal. Additionally, the SNR is further improved by increasing the modulation frequency, which is a consequence of the decreased 1/*f* noise [64], as shown in Figure 5. Another important advantage of our method is that this gives on-line access to the fluorescence-free Raman spectra with a minimal required user intervention, making the method more practical and less time consuming with respect to the standard SERDS methods. Furthermore, multi-channel detection compared with single-channel detection requires much shorter signal accumulation times, rendering the method suitable for real-time static background removal, especially in the presence of biological samples, which are normally photochemically labile.

### 3.4. Identification of Bladder Tumour Cells in Urine Samples

**Figure 6:**
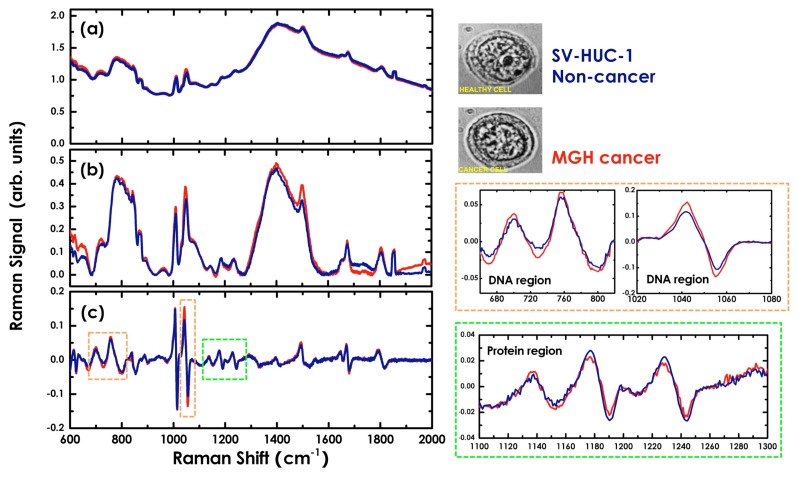
Mean spectra of normal (blue) and bladder cancer cells (red line) acquired with standard Raman spectroscopy (**a**), after polynomial baseline subtraction (**b**) and with modulated Raman spectroscopy (**c**). A zoom in the spectral regions between 660 and 820, 1020 and 1080 and 1100 and 1300 cm^−1^, for modulated Raman spectra, is also shown [65].

In this section, we demonstrate the application of our modulated Raman spectroscopy to the identification of normal human urothelial cells (SV-HUC-1) and cells derived from a recurrent human bladder tumour (MGH) [65]. Figure 6 displays a comparative overview of standard and modulated mean Raman spectra of SV-HUC-1 and MGH cells on 100 acquisitions. The laser power on the sample during signal acquisition was ∼10 mW. Each standard spectrum was collected with an integration time of 200 s. In the modulated approach, for each cell, 40 stacked spectra were recorded with an acquisition time of 5 s each while the laser wavelength was modulated with a modulation frequency of 40 mHz and a modulation amplitude of 60 GHz (corresponding to ∼0.2 nm at this wavelength). The 40 stacked spectra were on-line analysed by using the PCA method for fluorescence suppression, and modulated Raman spectra of the cells were obtained. In Figure 6, it is clear that standard Raman spectroscopydoes not allow the finest spectral details of the cells to be defined due to the presence of the strong fluorescence background. Conversely, in the modulated Raman spectra of SV-HUC-1 and MGH cells, the Raman peaks of the chemical constituents of the cells can be clearly observed. An overview of the identified cell signals and their corresponding vibrational assignment according to the literature is presented in Table 2. By comparing the relative intensities of the spectral peaks for modulated SV-HUC-1 and MGH spectra, we show that the SV-HUC-1 cells are characterized by relatively strong protein Raman peaks, in the spectral region 1100–1300 cm^−1^, which suggests greater concentrations of proteins in non-malignant cells. In contrast, the spectra from bladder cancer cells show a significant increase in the peaks pertaining to ring breathing modes in DNA bases, such as 727, 785, 1055 and 1578 cm^−1^, suggesting an increase in the DNA concentration in bladder cancer cells. For comparison, in Figure 6b, we show baseline-corrected Raman spectra where the Horiba Labspec 6 software package was used to subtract a 20th order polynomial from the standard spectra. This example highlights one of the main disadvantage of polynomial background fit. It is not possible for a mathematical algorithm to distinguish between Raman peaks and fluorescence background without any prior knowledge. This leads to a certain amount of sample-dependent background “leakage” onto the Raman spectra and, more importantly, a large variability between corrected spectra, even if the Raman component is identical.

The spectra acquired through the modulation technique present additional advantages when used in multivariate statistical analyses, such as PCA. By using PCA as a statistical tool, the individual spectra are analysed and separated into clusters for which the tightness of the clusters indicates the ability to discriminate cancer/non-cancer. Most importantly, PCA relies on a normal distribution of the noise. In the case of standard Raman spectroscopy, the noise cannot have a perfect normal distribution, because the spectral intensity measured is always positive, thus skewing the noise spread. In contrast, the Raman modulation spectra naturally oscillate between positive and negative values. The noise of the modulated Raman spectra can thus have a normal distribution [63].

**Figure 7:**
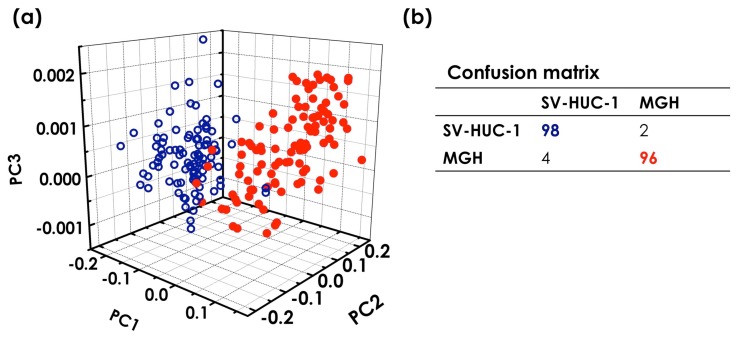
(**a**) 3D PCA scope plot of all individual modulated Raman spectra for 100 cancer (red dot) and 100 non-cancer (blue circle) cells; (**b**) confusion matrix.

The PCA applied to the modulated Raman spectra of 100 SV-HUC-1 and MGH cells delivers the 3D scatter plot shown in Figure 7a. The PCA data reduction method can be also used in a predictive way through cluster analysis, and the predictive performance can be checked using a leave one out cross-validation technique. In this case, we calculate the principal components of the whole dataset without one spectra. This forms a training set that is used to predict the classification of the left out spectra using the k-nearest neighbours method (k-NN) [91]. The k-NN approach associates an unknown spectra with the closest cluster in the training set (here, all other spectra previously measured). This cross-validation approach is used for each spectra in the set, and we construct a confusion matrix, reported in Figure 7b, which summarizes the correct and incorrect spectra classification. By analysing the diagonal terms of the confusion matrix, we can obtain a sensitivity and specificity up to 98% and 96%, respectively. The same analysis performed on standard Raman spectra provides a sensitivity and specificity of about 96% and 72%, respectively [65]. Similar results are reproduced after a few months' interval [92].

**Figure 8:**
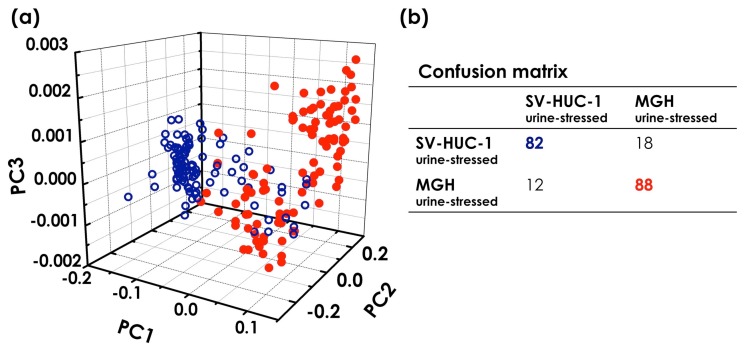
(**a**) 3D PCA score plot of modulated Raman spectra for 100 urine-stressed normal (blue circle) and 100 urine-stressed bladder cancer cells (red dot); (**b**) confusion matrix.

To mimic a scenario as close as possible to the clinical setting, the effects of urine have been also tested. We acquired 100 modulated Raman spectra of normal (SV-HUC-1) and bladder cancer (MGH) cells exposed to urine for six hours. PCA analysis performed on normal and cancer urine-stressed cells show distinct, separate clusters for each group of stressed cells [65]. The observed separation is probably due to DNA breakdown and protein damage in the urine-stressed cells. Thus, the chemicals in the urine can induce a detrimental effect on the collected cells, which is reflected in the PCA. However, PCA comparing the modulated Raman spectra of normal and tumour cells exposed to urine for six hours show distinct, separate clusters for the two stressed cell types (see Figure 8a). Even after six hours of exposure, the sensitivity and specificity are quite high: 82% sensitivity and 88% specificity. The observed decrease in terms of sensitivity/specificity, compared to the control sample (zero hours in urine), is probably due to the reduction of the signal-to-noise ratio in the Raman spectra induced by the urine stress. However, the same analysis performed on standard Raman spectra provides a sensitivity and specificity of about 76% and 71%, respectively [65].

### 3.5. Optimization of the Modulation Parameters for Cell Screening

In this section, we demonstrate a systematic approach to optimize the following key factors to improve the modulated Raman spectra for bladder cancer cell detection [67].

i Modulation amplitude, which refers to the range (*δν*) within which the laser wavelength was modulated while acquiring the Raman signal. This value should match the FWHM of the Raman peaks and the resolution of the spectrometer and, therefore, crucially depends on the analyte. Thus, we observed that by increasing the modulation amplitude from *δν* = 40 GHz to *δν* = 240 GHz and keeping constant the other parameters (sampling rate, modulation frequency, number of cycles and time constant), the corresponding SNR measured for MGH cells increases almost linearly. A completely different behaviour was observed for polystyrene bead samples, where a maximum SNR value is achieved at *δν* = 120 GHz [67]. This effect is due to different Raman cross-sections affecting the FWHM of the typical Raman peaks of polystyrene beads and MGH cells. For cell analysis, *δν* = 160 GHz corresponding to *δλ* = 0.32 nm was chosen, giving the minimum modulation amplitude that provides resolved Raman bands well within the laser mode-hop free region [67].
ii Modulation frequency, *f*, of the periodic laser wavenumber oscillations. We studied the effect in terms of SNR of a Raman signal for different modulation frequencies *f*, and we observed the presence of three regions [64], as shown in Figure 5. In the first region, the SNR increases almost linearly and then reaches a plateau. This trend can be easily understood: as the modulation rate increases, the signal-to-noise ratio increases as a consequence of the decrease in the 1/*f* noise. By further increasing *f*, the signal-to-noise ratio reaches a saturation plateau. This last trend can be attributed to the fact that at higher laser modulation frequencies, the acquisition time of the single spectrum is reduced, rendering the read-out-noise level of the CCD camera almost comparable to the peak intensity and, consequently, affecting the measurements. Finally, when the peak intensity is hindered by the read out noise level, the signal-to-noise ratio decreases again.
iii Sampling rate and time constant. The first one corresponds to the number (*n*) of acquisition steps per cycle; the second refers to the single exposure time (*δt*) for acquiring a Raman spectrum. By keeping constant the total integration time, we studied the Raman spectra of MGH cells for different sampling rates and time constants [67]. We observed that the sampling rate does not make a significant contribution to SNR variation, as long as three or more wavelengths are sampled, and, therefore, can be kept minimal [67]. Crucially, by increasing the time constant, an increase in SNR was observed, and the choice depends on the Raman cross-section of the analysed sample [67].
iv Total acquisition time, *T* = *nN_c_δt*, which corresponds to the product of the time constant, sampling rate and number of cycles (*N_C_*). By increasing the acquisition time and the number of cycles, of course, there is an increase of measured SNR in the modulated Raman signal.

**Figure 9:**
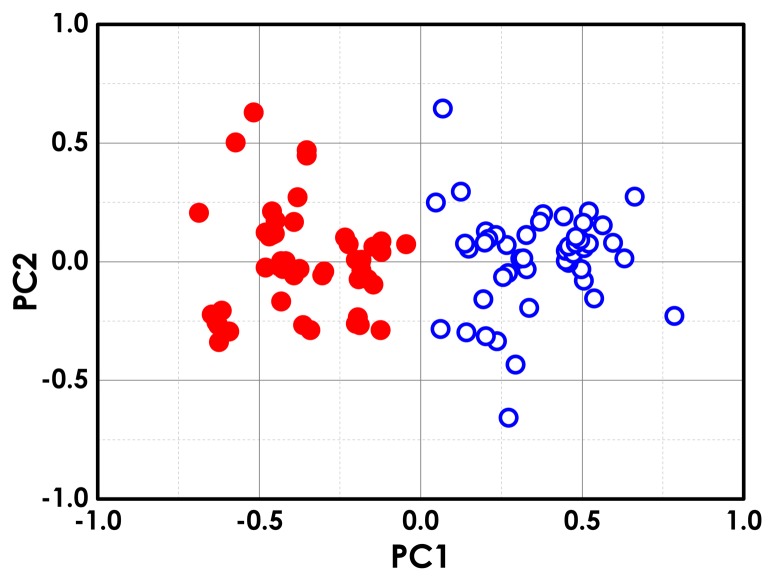
PCA score plot of modulated Raman spectra for 50 normal (blue circle) and 50 bladder cancer cells (red dot) acquired with a laser power of 200 mW, a modulation amplitude of 160 GHz, a sampling rate of three, a number of cycles of one, a time constant of 2 s and a total acquisition time of 6 s [67].

Thus, by keeping constant the sampling rate (n = 1), modulation amplitude *δν* = 160 GHz and modulation frequency *f* = 0.5 Hz, we studied the minimal total acquisition time, time constant and number of cycles required for modulated Raman spectra without affecting the discrimination ability between normal (SV-HUC) and bladder cancer (MGH) cells. We acquired 50 modulated Raman spectra from normal (SV-HUC) and 50 from bladder cancer (MGH) cells. The laser power on the sample was about 200 mW. We performed PCA analysis to measure the discrimination ability between cancer/non-cancer cells achieved by varying these three parameters (total acquisition time, time constant and number of cycles). We found that by increasing the number of cycles from two up to 10 increases the SNR of the Raman data without enhancing the discrimination ability. Additionally, we observed that the threshold SNR required to obtain efficient cancer/non-cancer discrimination can be obtained with *N_c_* = 1, n = 3 and a minimum time constant of 2 s, corresponding to a total acquisition time of 6 s, as shown in Figure 9 [67].

### 3.6. Towards Wavelength Modulation Raman Spectroscopy in Microfluidics

Microfluidic systems are becoming increasingly attractive in chemistry and biomedicine, as they allow for the miniaturisation of systems that are normally employed in those laboratories. Integrated Raman microscopy and microfluidic systems, “Raman-microfluidics”, have been already successfully applied in the analysis of materials from low-volume liquid media, especially when samples are rare and expensive (medical samples, forensic traces and pharmaceuticals) or environmental sample monitoring (water quality and biosensing). However, the efficiency of such a device plays an essential role in its possibility to succeed on the market and find a place in clinical practice.

**Figure 10:**
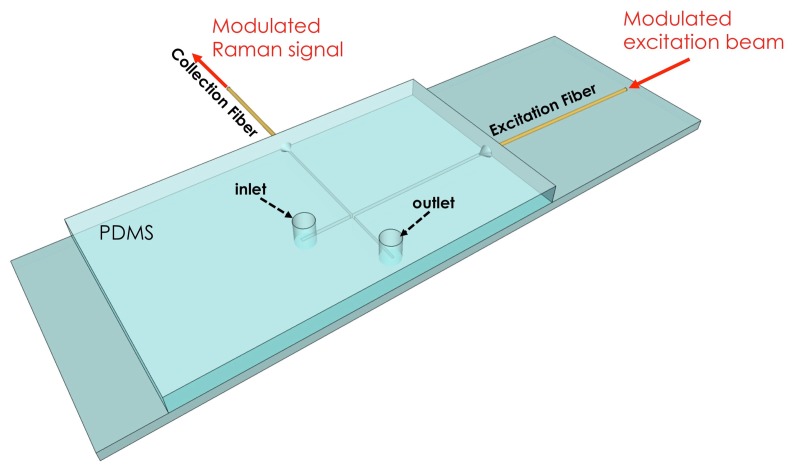
Scheme of the wave guide-confined Raman spectroscopy system [93]: a low OH multimode fibre (Polymicro Technologies, AZ, USA) of a core diameter of 200 μm delivers the modulated beam into the microfluidic chip for excitation; another fibre of the same specification was used to collect the Raman signal from the microfluidic channel and couple it into the spectrometer.

We recently demonstrated a wave guide-confined Raman spectroscopy (WCRS) approach, which implements fibre-optic-based Raman spectroscopy in microfluidic devices [93]. The microfluidic chip was fabricated in PDMS using soft lithography, and the scheme is shown in Figure 10a. This technique is scalable, easily incorporated with other microfluidic functional devices and is free from substrate background, as the detection wave guide is embedded into the microfluidic chip. Since fibres are used to excite and collect the Raman signal, the recorded Raman spectra contain fluorescence background from the fibres and the sample, limiting the effective detection sensitivity of the system. To overcome this issue, we implemented wavelength modulation in WCRS in order to eliminate the fluorescent background from the recorded Raman spectra [68]. By using urea solutions at different concentrations, we observed that by modulating the laser frequency at 45 mHz with an amplitude of *δν* = 40 GHz and a total acquisition time of 100 s, we can improve the SNR of the modulated compared to the standard Raman spectra (see Figure 8a). Additionally, measuring the variation of SNR with the urea concentration, we estimated the minimum detection limit of the system, which is the concentration at which the SNR becomes equal to unity [94], and the device sensitivity for standard and modulated Raman.

**Figure 11:**
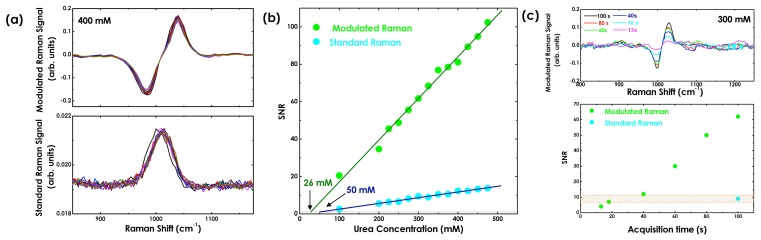
(**a**) Comparison of standard and modulated Raman spectra of a 400 mM urea concentration acquired in the wave guide-confined Raman spectroscopy configuration; (**b**) urea concentration vs. SNR for standard Raman (cyan) and modulated Raman (green) spectra; (**c**) variation of SNR in relation to the acquisition time for the Raman peak of urea at a 300 mM concentration in modulated Raman spectra [68]. The modulated Raman spectra of the 300 mM urea concentration acquired at different integration times are additionally shown.

In Figure 11b, it can be seen that the detection limit is only slightly smaller for modulated Raman spectroscopy, while the sensitivity of the device (slope of the curves in Figure 11b), which strongly depends on the spectra SNR influenced by background fluorescence, is seven-times increased for modulated Raman compared to that of the standard Raman spectra. This means that the resolution and robustness of WCRS for concentration prediction has significantly been enhanced with the implementation of the fluorescent suppression technique. Additionally, directly comparing the standard Raman spectrum obtained with an integration time of 100 s and the modulated Raman spectrum obtained with an integration time of only 20 s, we can clearly assess that the SNR values are comparable (see Figure 11c). This result demonstrates that the modulated Raman spectroscopy not only allows us to remove the fluorescence background, but also clearly improves the SNR, reducing the required acquisition time. Therefore, there is a great potential for WCRS to be used for developing alignment-free sensing optofluidic devices that can detect bioanalytes with minimal sample preparation.

The fabrication of a low-cost microfluidic device is one of the major challenges to its widespread use in biomedical diagnostics. Thus, we recently developed an alternative paper microfluidic compact Raman system. Paper microfluidics refers to dispensing with the use of soft elastomer-based microfluidics and resorting to an easy to fabricate and portable approach for point of care testing. In this method, the paper is patterned using techniques, such as ink-jet and wax printing, to write hydrophilic channels in the paper structure, guiding the liquid flow. However, as with bulk systems, the inherent background fluorescence of the paper substrate has meant combining Raman spectroscopy with paper microfluidics is not straightforward, requiring surface enhanced mechanisms. Wavelength-modulated Raman spectroscopy (WMRS) for analysis on a paper microfluidics platform allows us to suppress the background fluorescence of the paper and explore pharmaceutical analysis. Our data show that it is possible to discriminate between both paracetamol and ibuprofen and record nanomolar concentrations of each analyte [69].

## 4. Conclusions

One powerful technique for characterizing the chemical composition of biological systems is Raman spectroscopy. However, limited by the lower Raman cross-section and strong fluorescence background, complex and sophisticated instruments are usually required, so many commercial Raman spectrometers are restricted solely to optical laboratory use. In this paper, we summarized the state-of-the-art techniques for suppressing a strong fluorescence background and focused our attention on the wavelength-modulated Raman spectroscopy approach. We have demonstrated a novel modulation method that allows us to separate Raman scattering from fluorescence and to improve the spectral quality of the Raman data, even in comparison with other approaches. It can remove, in real time, a static background and render visible weak Raman features that are masked by the fluorescence background in the standard spectrum. In this work, we reviewed our modulation method, which provided correct classification of the Raman spectra of normal (SV-HUC) and bladder cancer (MGH) cells with high efficiency, without any prior data knowledge and minimal user intervention. Importantly, our approach leads to increased signal-to-noise ratios when compared with the ‘standard’ Raman and SERDS procedure, reducing acquisition time for data acquisition, such as a total acquisition time of only a few seconds being required, rendering the method more practical and less time consuming than other techniques. Finally, we demonstrated that integrated modulated Raman microscopy and microfluidic systems (wave guide-confined Raman spectroscopy and paper microfluidics) allow one to explore biological fluids/pharmaceutical analysis with high sensitivity and reduced cost. The obtained results suggest that this minimally-invasive optical technology has future potential for high-throughput cell screening.

## Figures and Tables

**Table 1:** Comparison between the presented fluorescence rejection methods in terms of advantages and disadvantages. Some examples of biomedical applications are additionally presented.

**Method**	**Pros**	**Cons**	**Biomedical applications**
**Mathematical approaches**	Low cost No set-up modification	Time-consuming Depend on user parameter choice Can cause data artifacts	Tissue diagnosis [17] Animal tissues [20] Identification of pathogenic microorganisms [24]
**Polarization gating**	Simple set-up modifications	Fails if fluorescence is not depolarized Not all Raman features can be detectedM	none
**Time-domain methods**	Very good fluorescence suppression	Complicate set-up modifications High cost Need for high-energy pulsed lasers Not useful when fluorescence lifetime is comparable to excitation pulse	Plant biology [39] Bone tissues [41] Brest tissues [42] Bladder and prostate tissues [43]
**Frequency-domain methods**	Good fluorescence suppression	Complicate set-up modifications High cost Require strong Raman signals	none
**Wavelength-domain methods**	Simple set-up modifications Medium cost Good fluorescence suppression	Raman spectra show a derivative shape	Human tooth [51] Animal tissues [61] Single biological cells [62]
**Modulated Raman spectroscopy**	Simple set-up modifications Medium cost Very good fluorescence suppression	Raman spectra show a derivative shape	Single biological cells [63] Cancer diagnosis [65, 67] Circulating tumor cells [66] In fiber study of biological fluids [68]

**Table 2:** Band assignment for the wavelength-modulated Raman spectra of SV-HUC-1 and MGHcells [2,66].